# GSA COVID-19 Task Force: Supporting Those in Practice

**DOI:** 10.1093/geroni/igaa057.2944

**Published:** 2020-12-16

**Authors:** Lisa Brown

**Affiliations:** Palo Alto, California, United States

## Abstract

The GSA COVID-19 Task Force developed multiple materials to assist older adults, those who care for them, and policy makers in understanding a variety of topics, including aging and immunity, ageism, the process of developing a vaccine, and how to social distance. Members of the group also undertook development of a decision aid on whether to engage in activities outside the home. The process of developing the decision aid according to Ottawa Research Institute guidelines will be explained.

